# Acknowledgment to Reviewers of *Pharmaceuticals* in 2021

**DOI:** 10.3390/ph15020184

**Published:** 2022-02-01

**Authors:** Pharmaceuticals Editorial Office

**Affiliations:** MDPI AG, St. Alban-Anlage 66, 4052 Basel, Switzerland

Rigorous peer-reviews are the basis of high-quality academic publishing. Thanks to the great efforts of our reviewers, *Pharmaceuticals* was able to maintain its standards for the high quality of its published papers. Thanks to the contribution of our reviewers, in 2021, the median time to first decision was 15 days and the median time to publication was 33 days. The editors would like to extend their gratitude and recognition to the following reviewers for their precious time and dedication, regardless of whether the papers they reviewed were finally published:
Abate, GiuliaLehmann, ChristianAbate, MarioLehnherr, HansjörgAbbasi, NooshinLehto, MikaAblain, JulienLeisz, SandraAbugri, Daniel A.Lemos, AgostinhoAcharya, ArpanLenci, ElenaAci-Sèche, SamiaLentini, GiovanniAdamczyk-Woźniak, AgnieszkaLeón-Rivera, IsmaelAdawy, AlaaLeopold, Jane A.Adhikari, Arijit A.Lesuisse, DominiqueAdhikari, Rajan DeepLesyk, Roman B.Adhikary, AmitavaLeung, BernardAdriaenssens, EvelienLezot, FredericAgamennone, MariangelaLi, Guang-LinAgarwal, PriyankaLi, JieAgbarya, AbedLi, LianboAgostino, MarkLi, Ruei-NianAguiar, SandraLiang, Po-HuangAguilar-de-Leyva, AngelaLiao, ChenghengAguilar-Zarate, PedroLiao, Jyh-feiAhmad, KhurshidLiao, Shu-ChuanAhmed, Atallah F.Liapi, CharisAhmed, WaseemLiaw, Chia-ChingAikins, DeaneLichtenauer, MichaelAksan, AysegülLiechti, Matthias EmanuelAlaibac, MauroLigat, GaetanAlam, MahboobLikar, RudolfAlbukhaty, SalimLim, Jeong-HoonAlbuquerque, HélioLim, Kwang SukAlcudia, AnaLim, Lee WeiAldewachi, HasanLim, SangminAl-Dujaili, EmadLima, BeatrizAlessio Ottaviani, AlessioLima, SofiaAlexandraki, KrystalleniaLin, Chen-YuanAlfinito, EleonoraLin, Chuen-FuAl-Hasan, Majdi N.Lin, FengAlhenc-Gélas, FrancoisLin, HaishuAli, Ibne K. M.Lin, Jer-AnAlkhateeb, AbedalrhmanLin, Kuo-ShyanAl-kinani, Ali AthabLin, RuweiAllam, Ayat A.Lin, Tz-FengAlles, Sascha R. A.Lin, Ying-HongAllott, LouisLinciano, PasqualeAlmeida, Adelaide P.Lindahl, LasseAlmoazen, HassenLinden, SaraAlonso, ConcepciónLinder, BenediktAlsaab, HashemLiu, BoAluicio-Sarduy, EduardoLiu, ChaoxingAlves, C. HenriqueLiu, JunyanAlves, FranciscoLiu, Po-YuAmarowicz, RyszardLiu, ShuangAmbrosio, LucaLlavero, FranciscoAmii, HidekiLo Nigro, LucaAmmazzalorso, AlessandraLo, Chih HungAna, CandalijaLoas, GwénoléAncuceanu, RobertLobo, JoaoAnderson, Peter M.Locarno, Silvia AliceAndersson, JanLochner, MartinAndrei, SandaLocker, FelixAndreotti, GiuseppinaLococo, Filippo MariaAndrianova, MariiaLoessner, HolgerAndroulakis, ‪X. MichelleLohans, ChristopherAngeletti, Paolo MatteoLok, Chun NamAngelino, DonatoLompardía, SilvinaAngelov, BorislavLongato, Giovanna BarbariniAngelova, AngelinaLonghini, FedericoAngrand, Pierre-OlivierLönnberg, HarriAngulo, JesusLopes, Carla MartinsAnmella, GerardLopes, Jose ManuelAnsari, Mohammad YunusLópez, ÓscarAnsari, RaisLópez-Hernández, Luz BereniceAnstead, Gregory M.López-López, MarisolAntal, DianaLötsch, DanielaAntal, IstvánLovrić, MarioAntoni, GunnarLoza-Mejía, Marco A.Antunes, Inês FLozano-Lozano, MarioApicella, AntonioLu, Chung-kuangArchibald, SteveLu, I-ChungArchie, Sabrina RahmanLu, ShaoyongArczewska, MartaLu, ShuiyuArdalan, MaryamLu, YiArdejani, MaziarLucas, Andrew T.Arias-Loste, María TeresaLuci, GiacomoArjunan, PachiappanLuethi, DinoArmakovic, Sanja J.Lungu, Claudiu N.Arnaboldi, SerenaLuo, Shi-ZhongArnet, IsabelleLuo, YongxiangArnhold, JürgenLupien, AndréanneArosio, DanielaLuvisetto, SiroArrivi, AlessioLuxen, AndréArsiccio, AndreaLuzina, OlgaArtalejo, Antonio R.Lyashchenko, SergeArteaga, Jesús FernándezLymberopoulos, AristotelisAsdaq, Syed M. B.Ma, EnboAshok, AjayMaas, RenkeAshour, Mohamed LotfyMabou Tagne, AlexAsperti, MichelaMaccagno, MassimoAstl, JaromirMaccallini, CristinaAsuero, Agustín G.Maccari, FrancescaAtanase, LeonardMacEwan, DavidAtanasova, MariyanaMachairiotis, NikolaosAttila, Papp LajosMaciej, StrzemskiAugustyniak, DariaMaciver, SutherlandÁvila-Flores, AntoniaMack, JohnAyub, RabiaMacNeill, AmyAziriova, SilviaMączyński, MarcinAzrad, MayaMadamsetty, Vijay SagarBabak, MariaMaeng, Han-JooBabon, Jeffrey J.Maffia, MicheleBaby, André RolimMagalhães, JoanaBąchor, RemigiuszMagdalena, Musuc AdinaBäcker, DanielMagdalena, WoźniczkaBácskay, IldikóMaggio, RobertoBadolati, NadiaMaggo, SimranBae, Ji-YeongMagiorkinis, Emmanouil N.Bae, Jung-WooMagrassi, LorenzoBaeza, AlejandroMagriplis, EmmanuelaBaffy, GyorgyMai, StefaniaBagyinszky, EvaMaioli, SilviaBak, AndrzejMajumder, Erica L.Bakshi, HamidMajumder, PoulamiBalayssac, DavidMakarska-Bialokoz, MagdalenaBalcells, SusanaMakwana, KamleshBalcerczak, EwaMalara, AlessandroBaldacci, FilippoMallandrich, MireiaBaldassano, SaraMallela, Shamroop KumarBaldassarre, Maria ElisabettaMallick, ‪PankajiniBaldisserotto, AnnaMalo De Molina, PaulaBalla, AndrásMałolepsza-Jarmołowska, KatarzynaBallinger, JimMalouf, CamilleBaltoumas, FotisMaltsev, Dmitrii S.Balusamy, Sri RenukadeviMancini, MaicolBanerjee, HirendranathMancuso, RaffaellaBanister, Samuel D.Manferdini, CristinaBano, GregorManfredi, FrancescoBansal, KuldeepManfredini, StefanoBansal, Kuldeep K.Mangalagiu, IonelBanti, Christina N.Mangge, HaraldBaranauskiene, LinaManini, PaolaBarbalexis, PanagiotisMannino, David M.Barbara, BreznikManolescu, Loredana Sabina CorneliaBarbasiewicz, MichalMansouri, KamelBarbieri, AntonioManzotti, GloriaBarbinta-Patrascu, Marcela-ElisabetaMaramai, SamueleBari, EliaMaranesi, MargheritaBarreiro, EstherMarazioti, AntoniaBarrero, María JoséMarchese, MariaBarrott, JaredMarchesini, MaurizioBartel, KarinMarciniec, KrzysztofBartels, DortheMarcocci, Maria ElenaBartholomä, Mark DanielMarco-Rius, IreneBartosinska, JoannaMare, AncaBasak, IndranilMarginǎ, DenisaBasco, LeonardoMargit, BalazsBassanini, IvanMarian, BrigitteBastiat, GuillaumeMarimuthu, ParthibanBasu, SambuddhaMarin, LuminitaBatrakova, Elena V.Marini, Herbert RyanBattistelli, CeciliaMarkiewicz, RoksanaBattistelli, MichelaMármol, InesBauckneht, MatteoMarotta, NicolaBaykov, Sergey V.Marques, M. MatildeBazan-Socha, StanislawaMarradi, MarcoBecker, SidneyMarrazzo, PasqualeBegines Ruiz, BelénMartens, JürgenBehbahani, HomiraMartha C., Rosales-HernándezBelluzzi, ElisaMartín Palma, M. ElenaBender, DirkMartinez Iglesias, OlaiaBenedetti, FrancescaMartínez-Hernández, JoséBenelli, RobertoMartinez-Navarrete, GemaBenfaremo, DevisMartinez-Useros, JavierBenhar, ItaiMartinez-Valladares, MariaBenlloch, MariaMartín-Gil, JesusBennardo, LuigiMartino, SabataBennett, James P.Martins, IvoneBenyhe, SándorMartins, José A.Benz, RudolfMartin-Saavedra, Francisco M.Berger, Daniela CristinaMartin-Sabroso, CristinaBergmann, RalfMaruf, Abdullah AlBergström, HelenaMarutescu, Luminita GabrielaBermudez, MarcelMascellino, Maria TeresaBernard, Marek K.Mašič, Lucija PeterlinBernardes, NunoMasieri, FedericaBernardini, GiuliaMassai, LaraBernardo, GanekoMassimi, MaraBernstein, Hans-GertMasson, JustineBernstein, NiritMassotte, DominiqueBertagnin, ChiaraMasternak, JoannaBertzbach, Luca DaniloMastrotto, FrancescaBessa, LucindaMatassini, CamillaBethanis, KostasMatejtschuk, PaulBezdetnaya-Bolotine, LinaMatesanz, Ana I.Bhagavathula, Akshaya SrikanthMathijssen, RonBhattacharya, SomanonMatijašić, GordanaBhawal, Ujjal KumarMatikonda, SiddharthBiagini, GiuseppeMatsubara, TakumaBianco, ArmandodorianoMattoscio, DomenicoBielenica, AnnaMattsson, SofiaBiernasiuk, AnnaMatulic, MajaBilewicz, AleksanderMatwijczuk, ArkadiuszBilia, Anna RitaMaugeri, AlessandroBillack, BlaseMaurea, NicolaBilliald, PhilippeMauricio, IsabelBilski, JanMaxim, CătălinBinda, ElisaMazet, RoselineBito, TomohiroMazhukin, DmitriiBivona, GiuliaMazumder, KishorBlack, DavidMazzone, PellegrinoBlackburn, JessicaMcCarthy, FlorenceBlanco-Salas, JoséMclean, BrendanBlazewicz, AnnaMcLean, GaryBlevins, James ErnieMd, ShadabBlomqvist, KimMeans, Robert T.Bochniarz, MariolaMeccariello, RosariaBoffano, PaoloMedina-Franco, José L.Bogár, FerencMegighian, AramBogdanov, PatriciaMegiorni, FrancescaBohinc, KlemenMehbub, Mohammad FerdousBojinova, RossianaMeléndez-Alafort, LauraBolás-Fernández, FranciscoMelnikova, NinaBolbasov, EvgeniyMelo-Carrillo, AgustinBolla, Pradeep KumarMenéndez-Arias, LuisBolzati, CristinaMenger, Fredric M.Bonfili, LauraMenghini, LuigiBönig, HalvardMenichelli, DaniloBonora, ElenaMennillo, ElviraBontempo, PaolaMerino Peláez, GraciaBoothello, RioMertlíková-Kaiserová, HelenaBora, AlinaMessina, AndreaBórawski, PiotrMessineo, DanielaBorota, AnaMessore, AntonellaBorrego-Sánchez, AnaMestre-Ferrandiz, JorgeBose, Rajendran J. C.Miastkowska, MałgorzataBotez, ElisabetaMicale, NicolaBougen-Zhukov, NicolaMichal, MalčekBouhlel, AhlemMichalska, KatarzynaBouropoulos, NikolaosMichel, Martin ChristianBousquet, GuilhemMichel, PiotrBovin, NicolaiMieczkowski, AdamBozic, JoskoMierzejewski, PawełBrachner, AndreasMiguel-Hidalgo, Jose JavierBradley, David S.Mika, DelphineBrągoszewski, PiotrMikstacka, RenataBranca, Jacopo Junio ValerioMilano, FrancescoBrandt, BurkhardMilanova, AneliyaBrandt, MarieMilanowski, Łukasz M.Branowska, DanutaMilardi, DaniloBraun, RalfMilavetz, BarryBrioli, AnnamariaMilczarek, MałgorzataBrisinda, GiuseppeMilella, LuigiBroecker, FelixMileva, MilkaBrogi, SimoneMilewski, SlawomirBrooks, AllenMiller, ElżbietaBrooks, Benjamin D.Mimura, ImariBrosens, ErwinMin, Sun-JoonBrown, Richard J. P.Minnelli, CristinaBrukner, IvanMiranda, MaríaBrulíková, LucieMircioiu, ConstantinBrullo, ChiaraMiricescu, DanielaBrüningk, SarahMironeasa, SilviaBruno, FedericaMisaka, TomofumiBrunt, TiborMisawa, TakashiBrüßler, JanaMishra, SaurabhBrycki, BogumilMiskulin, MajaBryniarski, KrzysztofMitani, ShoheiBrzezinski, MarekMitchell, Cassie S.Buccioni, MichelaMitra, AvishekBucekova, MarcelaMitsiou, DimitraBuchholz, MirkoMiyamae, YusakuBucolo, ClaudioMiyamoto, HirotakaBuda, Valentina OanaMlejnek, PetrBudai-Szűcs, MáriaModica De Mohac, LauraBūdienė, JurgaMognetti, BarbaraBudzevich, MikalaiMohanram, HariniBueno-Silva, BrunoMohr, AndreaBukhovets, Aleksandra V.Mojumdar, KamalikaBulk, EtmarMolgó, JordiBuratti, LauraMolina-Cerrillo, JavierBurchacka, EwaMolina-Leyva, AlejandroBurek, MalgorzataMoloudizargari, MiladBurge, Kathryn Y.Moltrasio, ChiaraBurghardt, KyleMonari, AntonioBurmester, AnkeMonchaud, DavidBurns, KatieMontaño, Gabriel A.Busardó, Francesco P.Montenegro, LuciaBusby, YanMontes, GuilhermeCaba, OctavioMonti, BarbaraCacciola, FrancescoMoon, Il SooCacciola, Nunzio AntonioMoosa, Mahdi MuhammadCaceres, SaraMorais, MauricioCacialli, PietroMorak-Młodawska, BeataCagliero, Cecilia LuciaMoreno, EstherCai, ShuoweiMoreno, SandraCalabrese, GiovannaMoretti, MatteoCalarco, AnnaMorganti, PierfrancescoCaltabiano, RosarioMorgat, ClémentCamargo, MarianaMorgese, Maria GraziaCammarata, Silvia MiriamMori, HiroyoshiCampagna, RobertoMori, MattiaCampanati, AnnaMorini, LucaCampello, PaulaMorozova, Vera V.Campos Rosa, Joaquín MaríaMosqueira, Vanessa Carla FurtadoCampos-Montiel, Rafael G.Mossialos, DimitrisCanales-Martinez, MargaritaMotamed, CyrusCanfield, ScottMota-Morales, Josué D.Cangemi, GiulianaMott, Derrick M.Cangkrama, MichaelMoyá, María LuisaCannataro, RobertoMozos, IoanaCannon, Ronald E.Mu, LinjingCapasso, RaffaeleMueller, CristinaCapece, MarcoMulier, Jan PaulCaputo, GregoryMüller-Vahl, Kirsten R.Carballo, RosaMulloy, BarbaraCarboni, LuciaMuñiz-Márquez, Diana B.Cardador-Martínez, AnabertaMuñiz-Ramirez, AlethiaCardoso, Susana M.Munteanu, Alexandra CristinaCardullo, NunzioMur, PilarCarmine, ColucciniMuraki, KatsuhikoCarnovali, MartaMuraleva, Natalia A.Carone, Benjamin R.Murdaca, GiuseppeCarr, Carolyn A.Murphy, NiallCarregal-Romero, SusanaMurthy, PreetishCarrella, SabrinaMusteata, MihaiCarroll, LaurenceMutsaers, Henricus A. M.Carvalho, CarinaNadǎş, George CosminCarvalho, CristinaNagy, BélaCasanova, Alfredo G.Nagy, ElodCasapullo, AgostinoNagy, MiklósCasaril, AngelaNakajima, AkiraCasertano, MarcelloNakajima, HidetoCasiraghi, AntonellaNakanishi, TakeoCasteleijn, MarcoNalepa, IrenaCastro-Torres, Rubén DaríoNalli, MariannaCatanzano, OvidioNamjoshi, SarikaCavallero, SerenaNapolitano, FilomenaCaviglioli, GabrieleNarcisa, MandrasCazorla-Luna, RaúlNaros, GeorgiosCebrián Castillo, RubénNasim, Muhammad JawadCen-Pacheco, FranciscoNasreddine, RoubaCepeda, JavierNassini, RominaCerchia, LauraNatarelli, LuciaCerra, BrunoNavarra, MicheleCervellati, FrancoNavarrete-Vazquez, GabrielČesla, PetrNawrot, BarbaraCespi, MarcoNazaruk, EwaCetinel, SibelNechifor, GheorgheChai, Tsun-ThaiNejman-Faleńczyk, BożenaChan, Kiat HwaNencka, RadimChang, Nai-JenNeng, NunoChanishvili, NinaNeo, Ana Maria GomezChao, Louis KuopingNesterkina, MariiaCharvet, Claude L.Neufurth, MeikChatelut, ÉtienneNeut, ChristelChatterjee, IshitaNg, Wei LongChaves-Martinez, Felipe JavierNicoara, SimonaChellakkan, BlessonNicolai, MarisaChen, Chuan-LinNicolas, CyrilChen, Chun-JungNicoletti, FerdinandoChen, DaiqinNicos, MarcinChen, Fen-ErNicosia, AldoChen, GangNiculae, DanaChen, HuiNiculae, MihaelaChen, Kuan-HsingNiculescu, LoredanChen, LechuangNieoczym, DorotaChen, Peng-JenNifli, Artemissia-PhoebeChen, QiNikolakaki, EleniChen, RenxunNikolett, KállaiChen, XiaoyanNikolova, BilianaChen, XuqiaoNiles, Richard M.Cheng, Hong ShengNilsen, AaronCheng, Juei-TangNilsen-Hamilton, MaritCheng, Jya-WeiNishi, KosukeCheng, Yuan-BinNishida, KoyoChetoni, PatriziaNissilä, EijaChia, SeanNitulescu, George MihaiChiarelli, LaurentNitulescu, GeorgianaChin, JenniferNitulescu, MihaiChiniforush, NasimNitzsche, BiancaChinnathambi, ShanmugavelNiu, BenChirgwin, John M.Noda, MasafumiChiriac, AuricaNogales, AitorChizhov, Alexander O.Nogara, PabloChmielarz, PiotrNoguchi, YoshihiroCho, Cheong-WeonNorbrega, FranklinCho, Kwang-HwiNotni, JohannesChoi, Min SikNovak Kujundžić, RenataCholewinski, GrzegorzNovikov, AlexanderChow, Aviva Shing FungNowak, AnnaChrobak, ElwiraNowak, DorotaChrzanowska, AlicjaNowak, PrzemysławChu, Shi-JyeNsooudi, Nasim NsooudiChu, XiangpingNycz, JacekChuang, Shih-YiNycz, Jacek E.Chubanov, VladimirO, Joo HyunChubarov, AlexeyObana, AkiraChupakhin, EvgenyObrenovich, Mark E. M.Cichero, ElenaObreza, AlešČierniková, SoňaOcampo-García, Blanca ElíCiesielska, AnnaOchi, ShinichiroCieslar, GrzegorzOduor, Joseph Michael Ochieng’Cieslewicz, ArturOgo, NaohisaCifelli, PierangeloOgorek, RafalCilia, GiovanniOh, JaeseongCimmino, GiovanniOh, Jae-WookCione, ErikaOhashi, NamiĆirić, AnaOhbuchi, ToyoakiCirmi, SantaOhlenschläger, OliverCitro, SimonaOhoka, NobumichiCiura, KrzesimirOhta, EtsuroClanchy, FelixOhta, ShinjiClassen, Carl FriedrichOhtsuka, TakashiClericuzio, MarcoOkamoto, MasakiCocco, StefaniaOkazaki, JojiCodacci-Pisanelli, GiovanniOkińczyc, PiotrColasante, ClaudiaOkino, NozomuColone, MarisaOkuda-Ashitaka, EmikoColuccia, MauroOkura, TsuyoshiComi, CristoforoOla, Antonius R. B.Conde, SilviaOlah, Neli KingaContardi, MarcoOlaru, Octavian TudorelContreras, Joey A.Olbryt, MagdalenaCoombs, MelanieOlchowik-Grabarek, EwaCooper, Robin LewisOlczyk, KrystynaCordani, MarcoOlennikov, DaniilCordeiro, Ana SaraOliveira, HélderCorreia, Margareta P.Olivier, Jean LucCorreia, Sónia C.Olucha Bordonau, Francisco EliseoCorsaro, CarmeloO’Meara, MatthewCortés-Benítez, FranciscoOmilusik, Kyla D.Cortes-Hernandez, PaulinaOo, AdrianCosma, PinalysaOpsenica, Dejan M.Costa, Anna MariaOrlova, AnnaCosta, RobertoOrnello, RaffaeleCozza, GiorgioOrsini, GiovannaCramer, Gwendolyn M.Ortega, Joseph ThomasCrisan, LuminitaOrtega, Miguel A.Crona, DanielOrtiz-Masiá, DoloresCrupi, PasqualeOrtuño-Costela, María Del CarmenCruz-Chamorro, IvanOrzelska-Górka, JolantaCsanádi, MarcellOstos Marcos, Francisco JoséCsapo, EditOstos, Francisco JoséCsík, GabriellaOstróżka-Cieślik, AnetaCsuk, RenéOthman, RahmehČukić, MilenaOtręba, MichałĆwikła, JarosławOtsuki, TakemiCwynar, PrzemyslawOzaita, AndrésCzaplewski, CezaryOzaltin, KadirCzernel, GrzegorzOzarowski, MarcinCzumaj, AleksandraPaavola, JereCzyrski, AndrzejPacifici, RobertaDa Pieve, ChiaraPacini, MatteoDa Silva, Inês VieiraPadnya, PavelDa Silva, Luís PintoPafundi, Pia ClaraDaci, ArmondPagano, BrunoDadachova, EkaterinaPagare, PiyushaDagnon, SoleyaPałasz, ArturDaher, João P. L.Palazón-García, RamiroDal Col, JessicaPalethorpe, Helen M.D’Alfonso, LauraPałka, Jerzy A.Dalisay, Doralyn S.Pall, EmokeDamelio, NicolaPalladini, GiuseppinaDamiani, GiovanniPalleria, CaterinaDamon, Lloyd E.Pallotti, FrancescoDanalev, DanchoPalma, PauloDande, RanadheerPalma, SilviaDanila, EdvardasPalmer, Barry R.D’aquila, PaoloPaluszczak, JaroslawDarbon, PascalPan, ChunDas, PoushaliPan, GuoyuDas, PradipPan, HouwenDasgupta, ShatavishaPan, Hung-ChuanDawen Yu, EstherPandey, SudhirDawoud, DaliaPandolfi, FabianaDay, Andrew S.Paola, BrunDayem, Ahmed AbdalPapachristodoulou, AlexandrosDe Ceballos, Maria L.Paradisi, FrancescaDe Deurwaerdere, PhilippeParaskeva, EfrosyniDe La Luz Garcia-Hernandez, MariaParisini, EmilioDe Los Rios, CristobalPark, Jong KookDe Marco, RossellaPark, Jun BomDe Martin, SaraPark, Jung-hyunDe Masi, LuigiPark, JunsooDe Melo Barbosa, RaquelPark, Ki-cheongDe Molina, Ana RamírezPark, Myoung-HwanDe Mora, FernandoPartheniadis, IoannisDe Moraes, JosuéPartyka, AnnaDe Mori, AriannaPaśko, PawełDe Noronha, CarlosPassero, FelipeDe Rosa, LuciaPasupuleti, VisweswaraDe Sousa-Coelho, Ana LuísaPatarrão, RitaDe Vita, DanielaPatruno, MarcoDeb, SubrataPattabiraman, Padmanabhan P.Defant, AndreaPatterson, JenniferDegl’innocenti, DonatellaPatyra, EwelinaDe-la-Casa-Almeida, MaríaPaudel, PradeepDelgado, Daniel RicardoPaula, EneidaDelisle Rodriguez, DenisPaulo, AlexandraDelso, IgnacioPaulsen, FrankDémuth, BalázsPaventi, GianlucaDengler-Crish, ChristinePavlov, DanailDenny, BillPawlik, AndrzejDermitzakis, Emmanouil V.Pedersen, Per AmstrupDescoutures, Jean-MichelPedraza Chaverri, JoséDevkota, Hari PrasadPeer, CodyDey, NandiniPeh, Hong YongDharmaraj, RajmohanPeinelt, ChristineDi Gennaro, FrancescoPeiris, DinithiDi Gianfrancesco, LucaPeitl, PetraDi Leva, Francesco SaverioPelageev, Dmitry N.Di Micco, SimonePellegrini, ManuelaDi Paola, RosannaPellico, JuanDi Pietro, PaolaPeluso, PaolaDi Primio, RobertoPeña, JavierDi Salle, AnnaPeng, ChuanqiDi Stefano, AntonioPeng, DunfaDiamond, GillPeng, DuoDikici, Betul AldemirPerdih, AndrejDilger, James P.Pereira, CarlaDiliën, HannePereira, Patrícia A.Dimas, KonstantinosPereira, Patrícia AlexandraDimić, DušanPeretto, GiovanniDingley, Andrew J.Pérez Pérez, MaríaDinic, JelenaPerez, Ramon LopezDinischiotu, AncaPérez, SalvadorDioguardi, Francesco S.Perez-Alvarez, L.Direito, RosaPérez-Villanueva, JaimeDiţu, Lia MaraPeriasamy, RameshDługaszewska, JolantaPerioli, LuanaDługosz, EwaPerkovic, Matea NikolacDo Carmo, SoniaPerła-Kaján, JoannaDobchev, Dimitar A.Perron, HervéDocea, Anca OanaPerry, Maria De JesusDoda, Sai ReddyPešić, MilicaDoi, HisashiPeters, Godefridus J.Dołowy, MałgorzataPeterson, GregoryDomínguez-Álvarez, EnriquePethő, ZoltánDominguez-Vías, GermanPetr, JanDomoki, FerencPetrova, GuenkaDonatella, DianaPettit, NatashaDonner, DavidePezzuto, FedericaDorotea, Muck-SelerPieczonka, Adam M.Dorożyński, PrzemysławPiedade, Ana PaulaDos Santos Nascimento, Luana BeatrizPiegza, MichałDosoky, NouraPike, AndrewDossena, SilviaPilicheva, BisseraDotsikas, YannisPilkington, LisaDoytchinova, IriniPina, SandraDragan, Ecaterina StelaPineda, BenjamínDremencov, EliyahuPinho, Brígida RibeiroDuarte, AidaPinto, DianaDucza, EszterPinto, Diana CláudiaDuda, PrzemysławPinto, EdgarDudhipala, NarendarPintus, GianfrancoDuma, Virgil-FlorinPinzi, LucaDurai, PrasannavenkateshPiotto, Stefano P.Duranti, AndreaPippa, NatassaDurocher, FrancinePisanu, ClaudiaDuskey, Jason ThomasPisklak, Dariusz MaciejDutta, PranabanandaPitucha, MonikaDux, MáriaPiva, RobertoDyakonov, VladimirPlanas, MartaDylst, PieterPlatania, ChiaraDzikiewicz-Krawczyk, AgnieszkaPlatella, ChiaraEar, JasonPlater, Michael JohnEdiriweera, Meran KeshawaPłotka-Wasylka, JustynaEhrens, AlexandraPobiega, KatarzynaEjaz, AsimPoddelsky, AndreyEl Hadi, HamzaPodgorski, Iva I.El Raey, MohamedPodlewska, SabinaEl-Aarag, Bishoy Y. A.Pogrmic-Majkic, KristinaEl-Beih, Ahmed A.Poku, Rosemary A.Eleftherianos, IoannisPolano, MaurizioElgeti, MatthiasPolimeno, LorenzoElkamhaw, AhmedPolyakov, NikolayEl-Sawy, EslamPomel, SebastienEl-Seedi, HeshamPonce-Vargas, MiguelElshabrawy, Hatem A.Ponterio, Rosina CelesteElvas, FilipePontremoli, CarlottaEndre Károly, KristófPopa, Mircea IoanEngidawork, EphremPopescu, Mihaela R.Epping, Eric A.Popović Đorđević, JelenaEriguchi, MasahiroPorcu, LucaEritja, RamonPoręba, MarcinErjavec, Gordana NedicPortman, NeilEscalante, JaimePoŝa, MihaljEscargueil, AlexandrePospíšil, JiříEsteves Da Silva, Joaquim C. G.Potaczek, Daniel P.Esteves, Catarina V.Pourié, GrégoryEvani, Shankar JaikishanPourquier, PhilippeEyun, Seong-IlPoyet, Jean-LucFabrizio, MontecuccoPozzi, CeciliaFalzone, LucaPradhan, Bhola ShankarFan, JianglinPrat, MariaFanciulli, GiuseppePraveschotinunt, PichetFarace, CristianoPreto, JordaneFaria, JulianaPrieto, ElenaFasolato, CristinaPrimožič, InesFattah, SarinjPriotti, JosefinaFedorowicz, Joanna DagmaraProchazka, RadekFeher, GergelyPronin, Alexander V.Felczak, KrzysztofProsekov, AlexandrFelgueiras, HelenaProvenzano, EugenioFendt, MarkusPrzybył, Jarosław L.Feng, Chia-HsienPrzybyłek, MaciejFernandes, Maria HelenaPuangmalai, NichaFernandes, Maria José Da SilvaPucci, MarziaFernandez Ochoa, AlvaroPucek, AgataFernández, LucíaPulito, ClaudioFernandez-Campos, FranciscoPuray-Chavez, Maritza N.Fernández-Gutiérrez, MarPushchina, EvgeniyaFerraz, RicardoPutta, AnjaneyuluFerreira, FernandoPyka, AlinaFerreira, GuilhermePytka, KarolinaFerreira, Paula M.Qian, MingxingFezza, FilomenaQu, BingqianFierascu, IrinaQu, XiaozhongFifere, AdrianQuadri, Syed A.Figini, MariangelaQuax, PaulFilipič, BratkoQueiroz, Maria EugéniaFink, MaxQuiroga, Adoracion GomezFioretti, AlessandraRabanal, FrancescFiorino, FerdinandoRadaram, BhaskerFirer, Michael A.Radchenko, Eugene V.Fischer-Fodor, EvaRadu, BeatriceFleszar, MariuszRaducan, AdinaFletcher, JaneRadwan, Mohamed OsmanFoerster, Kathrin I.Ræder, JohanFogassy, ElemérRaghavendra, AksharaFoksiński, MarekRagusa, AndreaFong, KarenRahman, Md. HabiburFonseca Vázquez, BegoñaRai, AkhileshFontaine-Vive, FabienRaina, SatishFornaguera, CristinaRaji, IdrisFortuna, AnaRajnarayanan, RajendramFoster, DavidRamalho, Maria JoãoFouda, AmrRamalho-Santos, JoãoFracasso, GiulioRamani, VijayFraguas-Sánchez, Ana IsabelRamos, CarmenFranken, Anton A. M.Ramos, PawełFrański, RafałRamos, Ricardo MartinsFrati, AlessandroRampanelli, ElenaFrick, LucianaRamsbeck, DanielFriščić, MajaRancan, FiorenzaFroissart, RémyRangel, Gabriel WFrolova, LiliyaRango, MarioFrustaci, Anna MariaRanzato, EliaFuentes, Màrius V.Rao, K. S. JagannathaFujimori, NaoRao, Kummara MadhusudanaFujimoto, AyatakaRao, MahadevFujisawa, KiyoshiRapeanu, GabrielaFujita, MorifumiRapino, CinziaFumagalli, LauraRapone, BiagioFumoto, ShintaroRapposelli, SimonaFurlong, FionaRaspanti, MarioFusco, Francesca RomanaRassias, GerasimosFusco, NicolaRatajczak, MagdalenaGábor, ParagiRateb, MostafaGać, PawełRather, GulamGackowski, DanielRaugei, GiovanniGagliardi, AgneseRavaioli, FedericoGaglione, RosaRavara, SofiaGalabov, AngelRavera, EnricoGalanis, AlexisRawat, ManmeetGałązkowski, RobertRay, PriyankaGaleazzi, RobertaRaza, AunGaletti, MariclaRazo, RodrigoGallardo, EugéniaReal Oliveira, Maria Elisabete C. D.Gallo, MarcoRecabarren-Gajardo, GonzaloGallo, MonicaRegiec, AndrzejGaman, Mihnea-AlexandruReid, St PatrickGamarro, FranciscoReinehr, SabrinaGambella, AlessandroReinhard, JacquelineGanesan, A.Reis, Sara S.Ganesan, KumarReiss, Lucinda V.Gangadaran, PrakashRejinold, SanojGangaraju, RajashekharRekawiecki, RobertGarcía-Quintanilla, MeritxellRembiałkowska, NinaGarcía-Rodríguez, NéstorRemedios Serrano, DoloresGarcia-Serrano, Jose L.Renukuntla, JwalaGareb, BahezResende, Jarbas M.Gargallo, RaimundoRey Rios, Ana MaríaGarrido, María JesusReynisson, JóhannesGarrudo, Fábio Filipe FereiraRezaie, JafarGaspar, AlexandraRial-Hermida, IsabelGatea, FlorentinaRibaudo, GiovanniGawalek, JolantaRicci, ClaudiaGayen, ManoshiRicciardi, SaraGazdag, GáborRichert, AgnieszkaGazerani, ParisaRichter, Sara N.Gazzaruso, CarmineRichter, VladimirGe, BaoxueRiegger, JanaGęgotek, AgnieszkaRiemma, GaetanoGemma, SandraRiera, AntoniGenovese, CarloRigalli, Juan PabloGenschmer, Kristopher R.Rimoldi, IsabellaGentile, CarlaRinaldi, CarmenGentile, Maria TeresaRishi, ArunGentilin, EricaRizzo, AlessandroGeorgescu (Serb), AlinaRoberto, MichelaGeorgescu, Simona RoxanaRocha, DjenisaGeorgiev, Georgi As.Rochani, AnkitGeorgieva, SimonaRodolfo, CarloGeorgiou, ChristosRodrigo-Muñoz, José M.Gericke, AdrianRodrigues, António SebastiãoGermanidis, GeorgiosRodrigues, Luis MonteiroGeronikaki, AthinaRodrigues, MárcioGerrard, GarethRodríguez López, María IsabelGessler, FlorianRodríguez, AmaiaGharaghani, SajjadRodriguez-Dorantes, MauricioGhiciuc, Cristina MihaelaRodriguez-Lorenzo, LauraGhilardi Lago, João HenriqueRodríguez-Yoldi, MaríajesúsGhimire, Bimal KumarRogachev, ArtemGiaccone, LuisaRogozea, LilianaGiacomini, AriannaRoh, SanghoGiampietro, LetiziaRojo, Maria AngelesGianferrara, TeresaRolla, GiovanniGibuła-Tarłowska, EwaRoman, Gustavo C.Gigliotti, CasimiroRomano, Giovanni LucaGill, Pritmohinder S.Romański, MichałGillings, Nic M.Romao, CarlGiorgakis, EmmanouilRomero, AlejandroGiovarelli, MirellaRomiti, Giulio FrancescoGiuliani, DanielaRomosan, Radu-StefanGiunchedi, PaoloRona, RobertoGiusti, IlariaRong, ShisongGladysheva, Inna P.Roosterman, DirkGlazar, IrenaRosa, AntonellaGleńsk, MichałRoseanu Constantinescu, AncaGligor, FeliciaRosenau, FrankGliwiński, MateuszRosic, GvozdenGobeil, Fernand JuniorRossi, Gemma Caterina MariaGóbi, SándorRothbauer, MarioGobin, IvanaRoy, BidishaGodlewska, MarlenaRoy, DipankarGolay, JoseeRoy, Saktimayee MitraGomes-da-Silva, Lígia C.Rozalski, MarcinGomha, SobhiRubino, IlariaGonçalves, Helena M. R.Rui, LixinGoncharov, Nikolay V.Rusek, MartaGonzalez Mariscal, IsabelRusic, DorisGonzález, Florenci V.Rusu, AuraGonzález, María EugeniaRuszkowska-Ciastek, BarbaraGonzalez-Alvarez, MartaRuzza, PaoloGonzález-Garcinuño, ÁlvaroRyabchikova, Elena I.González-Marrero, JoaquinRybka, JakubGonzález-Minero, Francisco JoséRybka, Jakub DaliborGonzález-Muñiz, RosarioRzepecka-Stojko, AnnaGonzález-Rodríguez, María LRzymowska, JolantaGonzález-Sálamo, JavierSacco, ElenaGonzalez-Sanchez, EsterSadok, IlonaGonzález-Zamora, EduardoSaez, CarmenGoralczyk, KatarzynaSagatova, AliaGorasiya, SamirSagnou, MarinaGoričar, KatjaSahgal, PranshuGorynski, KrzysztofSaied, Essa M.Goto, KenichiSakabe, MasahideGotor, Alicia AyerdiSakamoto, AtsushiGrabarek, Beniamin OskarSalim, MalindaGramignoli, RobertoSalin, Alexey V.Gramolelli, SilviaSalmi, MikaGranados-Principal, Sergio M.Salvadori, NicolaGrauslund, MortenSalzer, IsabellaGray, Michael D.Šamec, DunjaGrigoriev, FedorSamhan-Arias, Alejandro KhalilGrimaldi, ManuelaSampaio, RuteGrochocki, WojciechSamykutty, AbhilashGrochowicz, MartaSanawar, RahulGroome, JamesSánchez Pérez, IsabelGruber–Bzura, Beata M.Sanchez-Ballester, NoeliaGu, ZhenglinSánchez-Montalvá, AdriánGuazzelli, LorenzoSánchez-Ramos, CeliaGué, EmilieSandhu, KiranGueron, GeraldineSandi, CarmenGuglielmi, PaoloSandre, OliverGuidotti, ElisaSansone, AnnaGuillen-Grima, FranciscoSantana, Andrés GonzálezGuix, Francesc XavierSantarem, NunoGulbins, ErichSantos, PauloGumieniczek, AnnaSantos, RicardoGunaje, JayaramaSapio, LuigiGuo, XingjieSaponara, SimonaGupta, KuledeepSarang, ZsoltGupta, PrachiSaravanan, M.Gupta, PranavSardo, AngelaGutiérrez-Abejón, EduardoSarecka-Hujar, BeataHa, Ki-TaeSarraguça, MafaldaHadjikakou, SotirisSartorius, RossellaHafner, AnitaSathiyaseelan, AnbazhaganHak, EelkoSato, DaisukeHakamata, WataruSato, HideyukiHakim, Md AbdulSato, WakiroHałas-Wiśniewska, MartaSato-Bigbee, CarmenHallan, Supandeep SinghSaturnino, CarmelaHamaguchi, ShogoSavvateeva, ElenaHamman, JoiasSawabata, NoriyoshiHammerl, Jens AndréSayed, Ahmed M.Han, DongWoonSayre, Casey L.Han, FelicitySazonova, Margarita A.Han, LiScacco, SalvatoreHanafy, Nemany Abdelhamid NemanyScarpellini, EmidioHanai, Jun-ichiScarselli, MarcoHancu, GabrielSchaiquevich, PaulaHanganu, DanielaScheau, CristianHannan, Md. AbdulSchechtman, DeborahHansel, KatharinaSchenone, SilviaHansen, Hinrich P.Scheres, JacquesHanyuda, AkikoScherma, MariaHao, JinsongSchillaco, OrazioHaque, MainulSchiltz, Nicholas K.Haque, Md. MamunulSchirrmacher, RalfHaque, Muhammad R.Schlich, MicheleHarada, MamoruSchmitt, AntoninHaragus, HoriaSchmitt, SébastienHardonnière, KévinSchoenhagen, PaulHaro, IsabelSchohn, HervéHarris, Audray K.Schorzman, Allison N.Harrison, Paul H. M.Schreuder, HermanHasegawa, MorifumiSchröder, Heinz C.Haslwanter, DeniseSchwaminger, SebastianHasni, IssamSchwarz, PatrickHaspula, DhanushSciacca, Michele Francesco MariaHassan, AhmedScicali, RobertoHassan, ImtaiyazŚciskalska, MilenaHatae, NoriyukiScotti, MarcusHayashi, MikioScuteri, DamianaHaynes, JenniferSechi, Gian PietroHaynes, RichardSee Hoe, Louise E.He, LixiaSegale, LorenaHe, QiuqinSelvaraj, ChandraboseHecel-Czaplicka, AleksandraSelvaraj, GurudeebanHefferon, Kathleen L.Selvaraju, Ram KumarHeffeter, PetraSemerci, NihanHeger, LukasSempruch, CezaryHeitmar, RebekkaSena, ArmandoHenderson, BrandonSencanski, MilanHendrickx, SarahSenćanski, MilanHendrix, JelleSenel, MehmetHeneberg, PetrSentkowska, AleksandraHeng, NicholasSeong, Yeon-SunHenrot, PaulineSephton, Selena MilicevicHerczegh, PálSerafini, GianlucaHernández-Breijo, BorjaSerafino, AnnaluciaHerrero-Cervera, AndreaSerefko, AnnaHerzog, DavidSereno, DenisHesham R. El-Seedi, Hesham R.Serrano, Dolores R.Hesse, MortenSertić, MirandaHesselgrave, NatalieSerwer, PhilipHeydemann, AhlkeSethi, GautamHicks, Paul B.Seto, ShigekiHiemke, ChristophSetzer, William N.Hill, DuncanSevastre, BogdanHimanen, JuhaSgaragli, GiampietroHirano, AtsushiSgrignani, JacopoHirono, KeiichiShah, ShivangHirosato, KandaShakeel, FaiyazHo, Yi-ChengShan, Sze Wan SamanthaHohmann, JuditShankar, EswarHolcman, KatarzynaShankargouda, PatilHong, BoohwiShanmugam, GobinathHong, Sung NohSharafutdinov, Irshad S.Horhat, DeliaSharma, KavitaHorhat, Florin GeorgeSharon Lee, HsiaojuHorng, Chi-TingShavandi, AminHorton, JohnShave, StevenHorvath, DragosShenderovich, IlyaHosaka, KayokoSheng, YueHossain, Kaniz Fatima BinteShepard, Blythe D.Hossain, Md. SanowerSherer, NathanHou, HarveyShestopalov, Alexander M.Hour, Mann-JenSheu, Sheng-HsiungHoyos, PilarShi, HuidongHrast, MartinaShimoda, MichikoHrckova, GabrielaShin, Crystal S.Hryniewicka, AgnieszkaShin, Meong CheolHsia, Shih-MinShin, So JinHsiao, Chung-DerShinoda, KeiHsiao, Kuei-YangShuler, CharlesHsieh, Chien-MingShuvalov, OlegHsin, Kun-YiSiddique, Abu BakarHsing, Chung-HsiSiebert, Hans-ChristianHsu, Chung-YuanSieniawska, ElwiraHsu, Hao-JenSievers, Laura KatharinaHsu, Kai-WenSignore, GiovanniHsu, Sheng-MinSil, SusmitaHsu, Shu-ChingSilberzweig, JeffreyHu, RuifengSilva, EduardaHuang, Cheng-YangSilva, JoãoHuang, Hui-ChiSilva, Marcelo SousaHuang, Jiann-JyhSilvestre, SamuelHuang, KangSilvestri, GiovanninoHuang, Shu-PinSilvestris, NicolaHuber, ReneSimirgiotis, MarioHuclier, SandrineSimon, ThomasHuh, Joo YoungSimpson, AnnHung, AndrewŠindlerová, LenkaHung, Ming-SzuSinenko, Sergey A.Huot, Joshua R.Singh, KamalendraHussein, MaythamSingh, ManishHwangbo, CheolSingh, SandeepIacob, Andreea-TeodoraSingh, SarbjitIbarra, Luis ExequielSingh, Sitanshu S.Idoate, MiguelSingh-Bains, Malvindar K.Ientile, RiccardoSinha, DebottamIeraci, AlessandroSiopa, FilipaIglesias-Carres, LisardSirinian, ChaidoIgotti, MariaSiristatidis, CharalamposIlia, GheorgheSiskos, Michael G.Im, Dong SoonSitarz, RobertImam, Syed SarimSivalingam, PeriyasamyImhof, PetraSiwek, MarcinImran, SaimaSkoda, JanIoele, GiuseppinaSkrypnik, KatarzynaIonita, PetreSkuja, SandraIqbal, Muhammad ShahidSkupin-Mrugalska, PaulinaIriepa Canalda, IsabelSlanar, OndrejIruzubieta, PaulaŚliwa, PawełIshiai, MasamichiSlominski, AndrzejIshii, TakahiroŚmieszek, AgnieszkaIskra, MariaSmolarczyk, RyszardIslas-Jácome, AlejandroSnežana, PapovićIssa, SamarSobeh, MansourIto, ShingoSobiak, JoannaIvanov, IliyanSobieska, MagdalenaIvanov, MarijaSobotta, ŁukaszIvanović, MilenaSodano, FedericaIwasaki, TakashiSogawa, NorioIwata, KoichiSohn, Ju-TaeIyer, SoumyaSokullu, EsenIżykowska, KatarzynaSolano, FranciscoJ Edagwa, BensonSolomonov, InnaJaburek, MartinSommerburg, OlafJacquelin, SebastienSoni, ChetnaJafri, MohsinSoreq, LilachJagusiak, AnnaSoriano-Sarabia, NataliaJahan, SadafSoto-Cámara, RaúlJain, KeertiSotomayor, Camilo G.Jakobusic Brala, CvijetaSousa, Maria C.Jakubczyk, EwaSousa, Sérgio FilipeJakubek, MilanSowa, IreneuszJalilian, ElmiraSpallarossa, AndreaJamal, Qazi Mohammad SajidSpanakis, MariosJana, BimalSpriggs, Meg J.Janagam, Dileep R.Srivastava, PranayJancsó, GáborSroda-Pomianek, KamilaJanda, KatarzynaSroka, RonaldJaniri, DelfinaStanciu, Gabriela-DumitritaJanowski, MiroslawStanisz, BeataJanuszkiewicz-Lewandowska, DanutaStankov, KarmenJarocka-Karpowicz, IwonaŠtarha, PavelJasiewicz, BarbaraStark, Felicity C.Jaśkowska, JolantaStarzak, KarolinaJastrzębska-Więsek, MagdalenaStasi, AlessandraJedelská, JarmilaStasiolek, MariuszJeleń, MałgorzataStătescu, CristianJembrek, Maja JazvinšćakSteardo, LucaJenner, FlorienStearns, DuncanJensen, Andreas IngemannStefania, RacheleJensen, MikaelStefanović, NikolaJeon, JonghoStepien, Karolina M.Jeong, Jun-hoSterk, Geert JanJerin, AlešStorvik, MarkusJezierska, AnetaStrekowski, LucjanJia, ShangStringaro, AnnaritaJiang, GuangdeStudzinska-Sroka, ElzbietaJiang, ZiwenSubramani Paranthaman, BalasubramaniJianu, Dragos CatalinSuperti, FabianaJicsinszky, LászlóSurapaneni, Krishna MohanJijie, RoxanaSurguchov, AndreiJillella, DineshSutherland, Hamish S.Jiménez-González, GemaSvardsudd, KurtJiménez-Jiménez, Félix JavierŚwiątek, ŁukaszJin, Hyo-EonŚwiątek, PiotrJochum, ChristophŚwiątkiewicz, IwonaJohn Jervis, PeterŚwierczek, ArturJohnson, Dylan M.Swilam, NohaJohnston, Kelly L.Szacon, ElzbietaJohnstone, CameronSzafranski, KrzysztofJojima, TeruoSzeiffova Bacova, BarbaraJoksimović, NenadSzeliga, MonikaJones, PeterSzewczyk, KatarzynaJose, JineySzewczyk-Golec, KarolinaJoshi, SameerSznitowska, MałgorzataJukič, MarkoSzopa, AleksandraJuncan, Anca MariaTabanca, NurhayatJung, Jong-WhaTabaszewska, MałgorzataJuśkiewicz, JerzyTabernero-Duque, María-JesúsKabir, Mohammad FaujulTagad, HarichandraKadam, AvinashTaghibiglou, ChangizKadela-Tomanek, MonikaTakasawa, KenKadokawa, Jun-ichiTakemori, HiroshiKafarski, PawelTamkovich, SvetlanaKagawa, NatsukoTaneja, IshaKairemo, KaleviTapia, Alejandro A.Kalam, Mohd AbulTarasiuk, JolantaKalayda, GannaTarasova, Ol’Ga A.Kalinin, VladimirTardugno, RobertaKalinina, Elena V.Tatiparti, KatyayaniKällback, PatrikTavakoli, JavadKalló, GergőTavanti, FrancescoKalogiouri, NatasaTaylor, Ronald P.Kamiloglu, SenemTchetina, Elena V.Kaminski, Daniel M.Teixeira, RóbsonKamysz, ElżbietaTéllez-Valencia, AlfredoKanda, TatsuoTemviriyanukul, PiyaKandeel, MahmoudTerret, Marie-EmilieKanemura, NobuhiroTesarik, JanKang, CongBaoThanki, Anisha MahendraKang, Jung HunThomale, JürgenKang, Ju-SeopThompson, Dorothea K.Kaňková, KateřinaThuillier, RaphaëlKantminienė, KristinaTinelli, AndreaKao, Cheng-YenTircso, GyulaKaplan, BarbaraTiritan, Maria ElizabethKaradžić Banjac, Milica Z.Tischler, DirkKarakaidos, PanagiotisTkachenko, KirillKaraś, MagdalenaTodde, SergioKaravasili, ChristinaToh, Wei SeongKarazniewicz, MartaTomaszewska, EwaKarbownik, AgnieszkaTombelli, SaraKardos, JuliannaTomek, PetrKarnati, Konda ReddyTonelli, MicheleKarpetas, George E.Torre, Maria LuisaKasprzak, ArturTorrisi, SebastianoKassa, JiriTortorella, PaoloKatarzyna, MalarzTósaki, ÁrpádKato, KatsuhikoToscani, DeniseKato, NobutakaToti, KiranKatona, GáborTrâmbițaș, CristianKatsifis, AndrewTrapani, DarioKaushik, PrashantTreglia, GiorgioKawakami, MasanoriTretjakovs, PēterisKawakami, SusumuTricarico, DomenicoKawamura, FumioTroein, PerKawanami, DaijiTsagareli, Merab G.Kawczak, PiotrTsai, Chi-HaoKawiak, AnnaTseng, Chih-HuaKayano, Shin-ichiTsitsilianis, ConstantinosKayser, Reilly R.Tsunoda, MakotoKazakova, Oxana B.Tu, ZongcaiKearney, Mary-CarmelTucker, JulieKennon-McGill, StefanieTundis, RosaKenzaka, TsuneakiTuranli, BesteKepka, AlinaTurdo, AliceKeramidas, AngeloTurk, FlorianKerem, TeralıTyszka-Czochara, MalgorzataKersten, GideonTyurin, Anton P.Kertmen, AhmetTzanova, MilenaKesari, Kavindra KumarTzeng, Nian-ShengKhalifa, ShadenTzeng, Shu-LingKhammanivong, AliUbaldi, MassimoKhan, AndleebUkidve, AnvayKhan, Muhammad BashirUnione, LucaKhandaker, MorshedUthaman, SajiKhapchaev, AskerVáclavíková, RadkaKhongkow, MattakaVaher, MerikeKhurana, NamrataVaismoradi, MojtabaKijewska, MonikaValdés-Baizabal, CatalinaKikuchi, HidehikoVale, NunoKim, BohyungVan Den Berk-Bulsink, MariekeKim, DonghwaVan Dijk, LisetKim, HoonVan Dyk, MadeleKim, Hwa-JungVan Lith, SanneKim, Hye OneVanelle, PatriceKim, JaesungVaramini, PegahKim, JinhaeVari, Camil-EugenKim, Ki SuVarì, Maria RosariaKim, KibeomVarriale, SimonaKim, Ki-YoungVarvaresou, AthanasiaKim, Min-KyuVasarri, MarziaKim, Ryong NamVega-Holm, MargaritaKim, SoochongVélaz, ItziarKim, WootaeVelena, AstridaKimura, AtsushiVelez-Cruz, RenierKimura, Ken-ichiVelisek, LiborKimura, NobuyukiVella, Filomena MonicaKinthada, LakshmanakumarVenerando, AndreaKiselev, AntonVenkatesan, Jagadeesh K.Kishikawa, NaoyaVennerstrom, JonathanKishimoto, DaigaVentura, CéliaKiss, AlexiVenugopal, DhamodharanKiss, AnnaVerbanac, DonatellaKiss, CsongorVerma, RajniKitamura, YoshiakiVerona, GuglielmoKitano, YoshikazuVerstraete, AlainKlar, AgnesVerticchio, AliceKleeff, JörgViapiana, AgnieszkaKlein-Weigel, PeterVicaș, Laura GrațielaKlochkov, S. G.Vicidomini, CaterinaKlockiewicz, MaciejVilla, ChiaraKluczyk, AlicjaVilloutreix, BrunoKo, Byoung JoonVilović, MarinoKobayashi, YuichiroVincendeau, MichelleKobuchi, ShinjiVincenti, FlaminiaKoh, Jae ChulVincentini, OlimpiaKohler, James J.Virgilio, AntonellaKoike, HarukiVismara, ElenaKoivisto, AriVitale, AlessandroKokkinakis, Emmanouil N.Vitetta, LuisKokotou, Maroula G.Vladymyrov, MykhailoKolar, MitjaVlahopoulos, SpirosKolobov, Alexandr AlexandrovichVlainić, JosipaKondo, HiromuVo, GiauKonduri, SrihariVochita, GabrielaKönig, JörgVolnova, Anna B.Koninckx, Philippe R.Von Neuhoff, NilsKonstantinidis, GeorgiosVorob’ev, Mikhail M.Kontos, Christos K.Vorobyeva, AnzhelikaKoolman, LeonardVulf, Maria A.Kopach, OlgaWähälä, KristiinaKorfei, MartinaWakiguchi, HiroyukiKorlyukov, Alexander A.Wang, JunfengKose, EijiWang, ShuminKosowska, AgnieszkaWang, Yeng-TsengKostopoulos, IoannisWard, Kristen M.Kosutova, PetraWatanabe, EiichiKot, BarbaraWawruszak, AnnaKotek, JanWeaver, JolantaKotta-Loizou, IolyWęgrzyn, AlicjaKotzalidis, GeorgiosWęgrzyn, GrzegorzKotzalidis, Giorgio D.Wei, Kai-CheKourkoumelis, NikolaosWeick, Jason P.Kovalská, MáriaWeina, Peter J.Kowalska, JolantaWelling, MickKowalski, RadosławWells, DominicKrähenbühl, StephanWenzel, BarbaraKrasel, CorneliusWenzl, RenéKrasteva, NataliaWernly, BernhardKrause, FrankWesołowski, MarekKrawiec, PaulinaWestra, JohannaKreps, FrantišekWiatrak, BenitaKrishna, GokulWiedlocha, AntoniKrishnakumar, DevadasWilliams, E. MarkKrishnan, ShanmugarajanWilliams, FrederickKristl, AlbinWilliams, Noelle S.Krokidis, MariosWilson, BriceKronek, JurajWimalasinghe, RasangiKrukow, PawełWiniarska-Mieczan, AnnaKryshchyshyn, Anna P.Wińska, KatarzynaKuan, Yu-HsiangWitty, Michael J.Kuçi, SelimWłodarczyk, MarcinKucinska, MalgorzataWłodarczyk-Stasiak, MarzenaKudo, TakashiWojsyk-Banaszak, IrenaKudova, EvaWozniak, KatarzynaKudryavtsev, Denis S.Woźniak-Budych, MartaKuenze, GeorgWozniak-Knopp, GordanaKukina, T. P.Wright, ColinKulbacka, JulitaWu, Chia ChunKumamoto, EiichiWu, ChunyiKumar, DhirajWu, Qing-LiKumar, RavindraWu, RigumulaKumar, SanjayWu, Sheng-NanKumar, SuneelWurm, Jan-PhilipKung, Woon-ManXu, JinbinKunsági-Máté, SándorXue, JiajiaKuo, Chan-YenYadav, DhananjayKuo, Shu-JuiYadav, Dharmendra K.Kurakula, MalleshYamamoto, KazuhiroKurczab, RafałYang, Chien-ChungKurnia Anjani, QonitaYang, Jai-SingKwok, Hang Fai (Henry)Yang, JieKwon, JaeyulYang, Se-RanLa Clair, JamesYang, Wen-BinLa Motta, ConcettinaYaraki, Mohammad TavakkoliLa Regina, GiuseppeYélamos, JoséLa Rosa, PiergiorgioYin, JianŁabuz, PrzemysławYoon, Hyeun JoongLachenmeier, DirkYoshida, AkihiroLachman, JaromirYoshida, MasahitoLambertucci, CatiaYu, Jian Q.Lambrinidis, GeorgeYuan, MengLamy, EvelynYun, Hwi-YeolLancelin, Jean-marcZabad, Rana K.Land, BenjaminZacconi, FlaviaLandi, SilviaZagórska, AgnieszkaLang, DiZahm, Christopher D.Larre, IsabelZairov, RustemLarsen, Julie BrogaardZalevsky, ArthurLasoń, WładysławZamostny, PetrLatté, Klaus PeterZanella, IsabellaLaurent, MichaëlZang, LiqingLavandera, Jose LuisZarrintaj, PayamLavoie, Kathleen HoeyZawilska, Jolanta B.Lawal, Ismaheel O.Zeineldin, MohamedLayek, BuddhadevZeiss, RenéLazzaroni, Maria GraziaŻelaszczyk, DorotaLe Thuc, OphéliaZhang, DuoLe, Hoang-ThanhZhang, ErshuaiLe-Deygen, I. M.Zhang, LiqiangLee, Cheng-IZhang, RunLee, ChoonghoZhao, LinboLee, Chung-LinZhiponova, MiroslavaLee, HankiZhou, KunLee, Jong BongZhou, QingyuLee, Kwang-WonZhou, ShaoboLee, Kyung-AhŽilius, ModestasLee, Li-JenZimmermann, MaxLee, Min WookZingg, Jean-MarcLee, Min YoungZitko, JanLee, SangkilZorena, KatarzynaLee, Seung HwanZovko Končić, MarijanaLee, Su-JunZubiaur, PabloLeggio, Gian MarcoZugaza, José LuisLehman, Sarah


